# Neural Responses of Acupuncture for Treating Functional Dyspepsia: An fMRI Study

**DOI:** 10.3389/fnins.2022.819310

**Published:** 2022-05-02

**Authors:** Xiaohui Dong, Tao Yin, Siyi Yu, Zhaoxuan He, Yuan Chen, Peihong Ma, Yuzhu Qu, Shuai Yin, Xiaoyan Liu, Tingting Zhang, Liuyang Huang, Jin Lu, Qiyong Gong, Fang Zeng

**Affiliations:** ^1^Acupuncture and Tuina School/The 3rd Teaching Hospital, Chengdu University of Traditional Chinese Medicine, Chengdu, China; ^2^Acupuncture and Brain Science Center, Chengdu University of Traditional Chinese Medicine, Chengdu, China; ^3^International Education School, Chengdu University of Traditional Chinese Medicine, Chengdu, China; ^4^First Affiliated Hospital of Henan University of Traditional Chinese Medicine, Zhengzhou, China; ^5^Department of Radiology, Huaxi MR Research Center, West China Hospital of Sichuan University, Chengdu, China

**Keywords:** acupuncture, functional dyspepsia, default mode network, reward network, functional magnetic resonance imaging

## Abstract

Different acupoints exhibiting similar therapeutic effects are a common phenomenon in acupuncture clinical practice. However, the mechanism underlying this phenomenon remains unclear. This study aimed to investigate the similarities and differences in cerebral activities elicited through stimulation of CV12 and ST36, the two most commonly used acupoints, in the treatment of gastrointestinal diseases, so as to partly explore the mechanism of the different acupoints with similar effects. Thirty-eight eligible functional dyspepsia (FD) patients were randomly assigned into either group A (CV12 group) or group B (ST36 group). Each patient received five acupuncture treatments per week for 4 weeks. The Symptom Index of Dyspepsia (SID), Nepean Dyspepsia Symptom Index (NDSI), and Nepean Dyspepsia Life Quality Index (NDLQI) were used to assess treatment efficacy. Functional MRI (fMRI) scans were performed to detect cerebral activity changes at baseline and at the end of the treatment. The results demonstrated that (1) improvements in NDSI, SID, and NDLQI were found in both group A and group B (*p* < 0.05). However, there were no significant differences in the improvements of the SID, NDSI, and NDLQI scores between group A and group B (*p* > 0.05); (2) all FD patients showed significantly increased amplitude of low-frequency fluctuation (ALFF) in the left postcentral gyrus after acupuncture treatment, and the changes of ALFF in the left postcentral gyrus were significantly related to the improvements of SID scores (*r* = 0.358, *p* = 0.041); and (3) needling at CV12 significantly decreased the resting-state functional connectivity (rsFC) between the left postcentral gyrus and angular gyrus, caudate, middle frontal gyrus (MFG), and cerebellum, while needling at ST36 significantly increased the rsFC between the left postcentral gyrus with the precuneus, superior frontal gyrus (SFG), and MFG. The results indicated that CV12 and ST36 shared similar therapeutic effects for dyspepsia, with common modulation on the activity of the postcentral gyrus in FD patients. However, the modulatory pattern on the functional connectivity of the postcentral gyrus was different. Namely, stimulation of CV12 primarily involved the postcentral gyrus–reward network, while stimulation of ST36 primarily involved the postcentral gyrus–default mode network circuitry.

## Introduction

Functional dyspepsia (FD) is a common functional gastrointestinal disease (FGID) with clinical incidence ranging from 8 to 40% (Ghoshal et al., 2011; Mahadeva and Ford, 2016). The main clinical manifestations of FD are epigastric pain, epigastric burning, early satiety, and postprandial fullness, which cannot be attributed to organic and metabolic causes (Tack and Talley, 2013; Drossman, 2016). FD not only significantly affects the quality of life (QoL) of patients but also creates severe socioeconomic burden (Talley et al., 2006). Due to the complex etiology of FD, there is currently a lack of effective pharmaceuticals (Tack and Camilleri, 2018; Yamawaki et al., 2018; Tack et al., 2019), so non-pharmaceutical therapies are sought out by doctors and patients alike.

Acupuncture, as the most commonly used alternative and complementary treatment modality worldwide, has been accepted as an effective therapy for FD (Yang et al., 2020; Wang et al., 2021). A number of clinical studies have shown that acupuncture not only can relieve symptoms of dyspepsia but can also improve patient QoL and emotional states (Xu et al., 2006; Ma et al., 2012; Zeng et al., 2012; Yang et al., 2020). For example, our previous results indicated that genuine acupuncture can significantly improve QoL and symptoms of FD patients when compared to sham acupuncture and oral itopride (Ma et al., 2012). A data mining-based review found that more than 20 acupoints can be used for treating FD in clinical practice. Among them, *Zusanli* (ST36) and *Zhongwan* (CV12) are the most commonly used (Cao et al., 2016a,b). Despite the two points exhibiting treatment efficacy, their mechanisms in the treatment of FD remain to be further studied.

In the last decade, a number of neuroimaging studies have demonstrated that FD patients exhibit significant functional and structural alterations in multiple brain regions, including the frontal cortex, somatosensory cortex, postcentral gyrus, precuneus, and caudate tail (Zeng et al., 2009; Nan et al., 2014; Lee et al., 2016; Qi et al., 2020), and these abnormal functional activities can be regulated by acupuncture, to some degree (Zeng et al., 2012; Chen et al., 2021). For example, a previous functional MRI (fMRI) study found that acupuncture at *Weishu* (BL21) and *Zhongwan* (CV12) can modulate the disrupted functional connectivity (FC) between the insula and other brain regions in rat models of FD (Chen et al., 2021). Our previous studies also indicated that needling acupoints on the stomach meridian could significantly reduce abnormally high glucose metabolism in the homeostatic afferent network of FD patients and that this regulatory effect is different from needling sham acupoints and from needling acupoints not used for treating FD (Zeng et al., 2012, 2015). However, the question of whether different acupoints with similar therapeutic effects share similar influences on brain function in patients with FD has not been explored in previous studies.

Therefore, on the basis of verifying similar clinical effects of ST36 and CV12 in the treatment of FD, our study aimed to 1) observe the influence of acupuncture on brain functional activities of all FD patients using fMRI, and attempt to determine potential target brain areas related to acupuncture efficacy, and 2) investigate the effects of needling at ST36 and CV12 on FC of the target brain region, in order to explore the mechanism(s) governing similar effects exhibited by different acupoints.

## Materials and Methods

This was a randomized controlled neuroimaging trial. The FD patients were recruited from the campus of Chengdu University of Traditional Chinese Medicine (CDUTCM) and the Digestive Department of the Affiliated Hospital of CDUTCM between January 2016 and May 2018. All patients were diagnosed by clinicians in the Digestive Department of the Affiliated Hospital of CDUTCM.

This study was performed according to the principles of the Declaration of Helsinki (Version Edinburgh 2000). The study protocol was approved by the Ethics Committee of the Affiliated Hospital of CDUTCM (No. 2014KL-028) and registered at the Clinical Trial Registry (registration number: ChiCTR-IOR-15006402).

### Participants

Patients were enrolled if they fulfilled all of the following inclusion criteria: (1) were aged 18 to 45 years; (2) were right-handed; (3) matched the Rome III diagnosis criteria for FD; (4) had not taken any gastrointestinal drugs or received acupuncture treatment for at least 15 days before entering the study; and (5) provided assigned informed consent. Patients were excluded if they (1) were pregnant or lactating; (2) had a history of head trauma with loss of consciousness or gastrointestinal surgery; (3) were currently taking drugs promoting gastrointestinal dynamics; (4) had any contraindications to acupuncture; or (5) had any MRI contraindications, such as pacemakers, fixed metal dentures, or severe claustrophobia.

After an initial 2-week baseline evaluation, 38 eligible patients were randomly assigned to two groups using a computer-generated randomization sequence. The randomization information was concealed from the researchers until the completion of statistical analysis. Patients were blinded to the group assignment.

### Acupuncture Intervention

**FIGURE 1:**
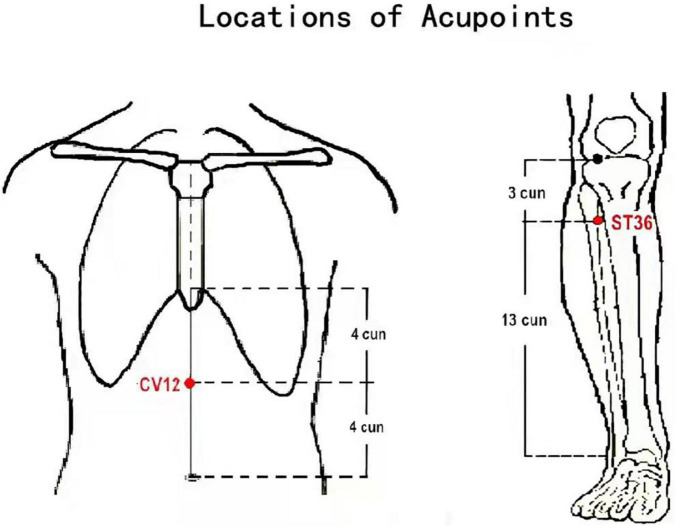
Acupoint locations of He-Mu point combination. *Zhongwan* (CV12): on the anterior median line of the upper abdomen, 4 cun above the navel. *Zusanli* (ST36): on the anterior lateral side of the shank, 3 cun below *Dubi* (ST35), one horizontally placed finger distance lateral to the anterior border of the tibia.

The acupoint used for group A was *Zhongwan* (CV12), while the acupoint used for group B was *Zusanli* (ST 36). The locations of the acupoints are shown in Figure 1. Acupuncture treatment was administered by two licensed acupuncturists with more than 6 years of clinical experience and who had received specialized acupuncture training in the selection of acupoints and standard acupuncture operating procedures. Manual acupuncture treatment was administered using disposable sterile filiform needles (25–40 × 0.25 mm, Huatuo Medical Instrument Co., Ltd., Jiangsu, China). The acupuncture treatment protocol used is outlined in our previous study (Yin et al., 2017). All patients received five acupuncture treatments per week for 4 weeks.

### Outcome Measurement

The Symptom Index of Dyspepsia (SID) and the Nepean dyspepsia index (NDI) were used to assess the efficacy of acupuncture for treating FD. The SID focused on the four chief symptoms of FD: postprandial fullness discomfort, early satiety, epigastric pain, and epigastric burning. Each symptom was graded as follows: asymptomatic (0 points), mild (1 point), moderate (2 points), or severe (3 points; Ma et al., 2012). The NDI is composed of the Nepean Dyspepsia Symptom Index (NDSI) and Nepean Dyspepsia Life Quality Index (NDLQI) (Talley et al., 1999). The NDSI evaluates the clinical symptoms of patients by measuring the frequency, intensity, and level of discomfort for 15 upper gastrointestinal symptoms. The NDLQI is an FD-specific questionnaire to assess patients’ QoL from four dimensions, including interference (13 items), knowledge/control (7 items), eat/drink (3 items), and sleep/disturb (2 items). Furthermore, the Self-Rating Anxiety Scale (SAS) (Zung, 1971) and Self-Rating Depression Scale (SDS) (Zung et al., 1965) were used to evaluate the emotional states of patients. The SID, NDSI, NDLQI, SAS, and SDS were measured at baseline and the end of the treatment.

### Functional MRI Scan

All patients received MRI scans at baseline and the end of the treatment. MRI data were acquired with a 3.0-T magnetic resonance scanner (Siemens, Munich, Germany) at Huaxi Magnetic Resonance Research Center, West China Hospital of Sichuan University, Chengdu, China. The scanning procedure contained a localizer, a high-resolution three-dimensional T1-weighted imaging (3D-T1WI), and a blood oxygenation level-dependent fMRI (BOLD-fMRI). According to previous studies, resting-state fMRI (rsfMRI) signals can vary when awake versus asleep, while the arousal level is closely related to rsfMRI signals at the sensorimotor region (Gu et al., 2019). Therefore, patients were told to maintain wakefulness during the scan to avoid the effect of falling asleep on brain activity in this study.

The scanning parameters were as follows: 3D-T1WI: repetition time (TR)/echo time (TE) = 1,900/2.26 ms; slice thickness = 1 mm; slices = 176; matrix size = 256 × 256; field of view (FOV) = 256 × 256 mm^2^. BOLD-fMRI: TR/TE = 2,000/30 ms; flip angle = 90°; slice number = 30; matrix size = 64 × 64; FOV = 240 × 240 mm^2^; slice thickness = 5 mm; total fMRI scans = 180, with the functional scan lasting 6 min.

### Statistical Analysis

#### Clinical Data

Data analysis was performed using SPSS 22.0 statistic software package (IBM Corp, Somers, NY, United States). Independent-samples *t*-test, Mann–Whitney U-test, and chi-square tests were applied to compare the baseline demographic and clinical characteristics of FD patients. A paired *t*-test or Wilcoxon signed-rank test was applied to compare within-group differences, and analysis of covariance (ANCOVA) was applied for between-group analysis. Correlation analysis was performed using Pearson’s correlation analysis. A *p*-value < 0.05 was considered statistically significant.

#### Functional MRI Data

##### Data Preprocessing

The functional BOLD data were preprocessed using Data Processing Assistant for Resting-State fMRI (DPARSF) software^1^ in MATLAB (MathWorks, Inc., Natick, MA, United States). During the preprocessing phase, the first 10 timepoints were discarded to avoid instability in initial MRI signals, and slice-timing correction, head motion estimation, and realignment were performed. Then images were segmented and coregistered to each patients’ high-resolution T1 scan and normalized to the standard Montreal Neurological Institute (MNI) template. After that, images were smoothed with a Gaussian kernel of 6-mm^3^ full width at half maximum (FWHM) and band-pass filtered with a frequency window of 0.01–0.08 Hz. Patients with excessive head motion [with mean frame-wise displacement greater than 0.5 mm (Power et al., 2012)] were excluded from the analysis.

##### Amplitude of Low-Frequency Fluctuation and Functional Connectivity Analysis

After preprocessing, the amplitude of low-frequency fluctuation (ALFF) was first calculated using DPARSF. Paired *t*-test was used to assess the within-group differences of ALFF in all FD patients. Gaussian random field (GRF) correction was used, and voxel-level *p* < 0.005 and corrected cluster-level *p* < 0.05 were considered statistically significant. Next, correlation analysis of the ALFF change values and clinical improvement values of all FD patients was performed to obtain the brain areas associated with acupuncture efficacy. Finally, the regions that were most significantly correlated with clinical improvement value were selected as regions of interest (ROIs) for FC analysis. The paired *t*-test was performed to investigate the functional alterations of FD patients before and after acupuncture treatment in each group. GRF correction was made, and voxel-level *p* < 0.05 and corrected cluster-level *p* < 0.05 were considered statistically significant.

## Results

Five participants were excluded due to excessive head motion (mean framewise displacement > 0.5 mm) during imaging. Thirty-three FD patients (18 in group A and 15 in group B) were included in the final clinical data and fMRI data analysis.

### The Baseline Characteristics of the Two Groups

The baseline characteristics of patients in the two groups are displayed in Table 1. As shown in Table 1, except for age, there was no significant difference in baseline characteristics between these two groups (*p* > 0.05; Table 1).

**TABLE 1:** The baseline characteristics in two groups.

Characteristic	Group A (*n* = 18)	Group B (*n* = 15)	Statistical value	*p*-Value
No. of women, n (%)	12 (66.67%)	13 (86.67%)	1.782	0.182
Age (years), mean ± SD	22.78 ± 1.63	21.20 ± 2.40	2.243	0.032*
BMI, mean ± SD	19.09 ± 1.48	19.70 ± 1.96	–1.030	0.311
Course of disease (M), mean ± SD	42.83 ± 26.15	33.60 ± 19.18	1.136	0.265
SID score, mean ± SD	3.89 ± 1.41	3.80 ± 1.42	–0.149	0.882
NDLQI score, mean ± SD	77.58 ± 8.41	74.60 ± 11.43	0.863	0.395
NDSI score, mean ± SD	45.67 ± 17.27	41.93 ± 14.07	0.671	0.507
SAS score, mean ± SD	40.32 ± 7.68	43.67 ± 9.77	–1.104	0.278
SDS score, mean ± SD	41.90 ± 9.11	45.18 ± 10.29	–0.971	0.339

BMI, body mass index; NDLQI, the Nepean Dyspepsia Life Quality Index; NDSI, the Nepean Dyspepsia Symptom Index; SAS, Self-Rating Anxiety Scale; SDS, Self-Rating Depression Scale; SID, Symptom Index of Dyspepsia. *p < 0.05.

### The Therapeutic Effects in the Two Groups

The within-group analyses showed that a significant increase in NDLQI scores and a significant decrease in SID scores, NDSI scores, SAS scores, and SDS scores were found in both group A and group B after acupuncture treatment (*p* < 0.05; Table 2).

**TABLE 2:** Comparison of the therapeutic effects between group A and group B.

Items	Group A					Group B					*F*-value	*p*
	Pre	Pos	Pos–Pre	*Z*-value	*p*	Pre	Pos	Pos–Pre	*Z*-value	*p*		
SID score	3.89 ± 1.41	1.94 ± 0.54	−1.94 ± 1.47	–3.573	0.000**	3.80 ± 1.42	1.20 ± 1.08	−2.60 ± 1.45	–3.427	0.001*	1.416	0.243
(mean ± SD)												
NDLQI score	77.58 ± 8.41	89.93 ± 5.81	12.35 ± 8.32	–3.636	0.000**	74.60 ± 11.43	88.88 ± 11.56	14.28 ± 8.47	–3.408	0.001*	1.224	0.277
(mean ± SD)												
NDSI score	45.67 ± 17.27	20.17 ± 10.14	−25.50 ± 13.87	–3.724	0.000**	41.93 ± 14.07	14.00 ± 14.48	−27.93 ± 14.92	–3.352	0.001*	0.988	0.328
(mean ± SD)												
SAS score	40.32 ± 7.68	34.72 ± 7.13	−5.60 ± 9.86	–2.329	0.020*	43.67 ± 9.77	34.00 ± 8.77	−9.67 ± 6.28	–3.297	0.001*	0.894	0.352
(mean ± SD)												
SDS score	41.90 ± 9.11	36.04 ± 8.43	−5.86 ± 10.58	–2.509	0.012*	45.18 ± 10.29	34.67 ± 8.67	−10.52 ± 9.13	–3.109	0.002*	0.349	0.559
(mean ± SD)												

*NDLQI, the Nepean Dyspepsia Life Quality Index; NDSI, the Nepean Dyspepsia Symptom Index; SAS, Self-Rating Anxiety Scale; SDS, Self-Rating Depression Scale; SID, Symptom Index of Dyspepsia.*

*p < 0.05; **p < 0.001.

The between-group analyses showed that there were no significant differences in the improvements of the SID scores, NDSI scores, NDLQI scores, SAS scores, and SDS scores between the two groups (*p* > 0.05; Table 2).

### Cerebral Activity Changes Induced by Acupuncture Treatment in Both Groups

#### The Amplitude of Low-Frequency Fluctuation Changes in All Functional Dyspepsia Patients

**FIGURE 2:**
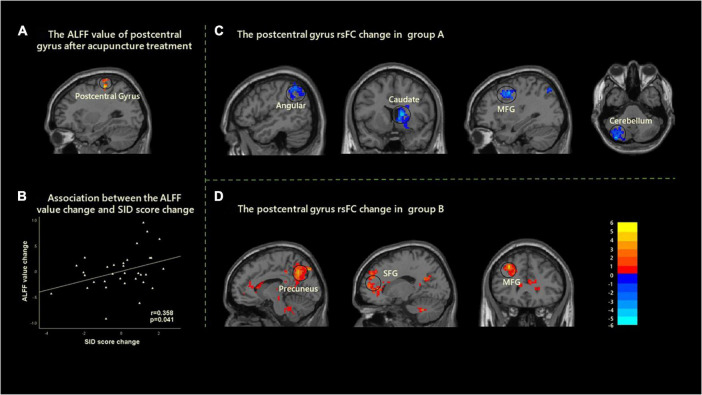
Cerebral activity changes induced by the acupuncture stimulation in two groups. **(A)** A significantly increased ALFF was found in the left postcentral gyrus after acupuncture treatment. **(B)** Correlation analysis showed that the change of ALFF value in left postcentral gyrus and the improvement of SID were positively correlated with age and gender as covariates (*r* = 0.358, *p* = 0.041). **(C)** After acupuncture treatment, the decreased rsFC between the left postcentral gyrus with angular, caudate, MFG, and cerebellum were found in group A. **(D)** After acupuncture treatment, the increased rsFC between the left postcentral gyrus with precuneus, SFG, and MFG was found in group B. ALFF, amplitude of low-frequency fluctuation; rsFC, resting-state functional connectivity; MFG, middle frontal gyrus; SID, Symptom Index of Dyspepsia; SFG, superior frontal gyrus.

After acupuncture treatment, significantly increased ALFF values in the left postcentral gyrus were found in all FD patients. Moreover, the ALFF value changes in the left postcentral gyrus were significantly related to the improvements of SID scores with age and gender as covariates (*r* = 0.358, *p* = 0.041, uncorrected) (Figure 2).

#### The Resting-State Functional Connectivity Changes of Postcentral Gyrus in the Two Groups

The left postcentral gyrus was selected as the ROI to investigate the FC changes of FD patients in group A and group B. Within-group comparisons demonstrated that there were significantly decreased resting-state FC (rsFC) between the left postcentral gyrus and angular gyrus, caudate, middle frontal gyrus (MFG), and cerebellum after treatment in group A. In addition, there were significantly increased rsFC between the left postcentral gyrus and precuneus, superior frontal gyrus (SFG), and MFG after treatment in group B (Figure 2 and Table 3). However, there was no significant correlation between FC and clinical efficacy in group A and group B.

**TABLE 3:** The differences of the left postcentral gyrus rsFC in each group.

ROI	Group	Cluster regions	L/R	Cluster sizes	Peak MNI			T
					*x*	*y*	*z*	
L_Postcentral Gyrus	Group A	Angular	R	280	49	−52	49	−3.74
		Caudate	R	274	15	9	12	−4.86
		Middle frontal gyrus	R	245	33	12	48	−4.42
		Cerebellum	L	338	−36	−81	−39	−5.51
	Group B	Precuneus	L	791	−10	−61	42	6.70
		Superior frontal gyrus	R	854	17	48	35	5.00
		Middle frontal gyrus	R	307	−30	30	42	6.37

*rsFC, resting-state functional connectivity; MNI, Montreal Neurological Institute; ROI, region of interest.*

## Discussion

The current study focused on the potential central mechanism of different acupoints sharing similar therapeutic effects for FD. The results demonstrated that needling at both CV12 and ST36 could elicit activity changes in the postcentral gyrus response, although their response patterns were relatively different. Needling at ST36 mainly affected the postcentral gyrus–default mode network (DMN) circuitry, while needling at CV12 mainly aroused the postcentral gyrus–reward network (RN) circuitry.

### The Similarities in Cerebral Responses Elicited by Acupuncture in Both Group A and Group B

In this study, the clinical observations indicated that needling at either CV12 or ST36 could significantly decrease SID scores, NDSI scores, SAS scores, and SDS scores and could increase NDLQI scores and that there were no significant differences in the improvements of variables mentioned above. The results indicated that both CV12 and ST36 were effective for improving symptoms, QoL, and emotional status of FD patients and that the therapeutic effects of both acupoints were similar. The current results were consistent with previous studies, which showed the effectiveness of both acupoints in the treatment of FD (Ma et al., 2012; Sun et al., 2021a). In addition, previous studies found that acupuncture treatment provided significant relief to gastrointestinal symptoms in comparison with FD patients awaiting acupuncture treatment (Chung et al., 2019). Therefore, performing acupuncture at CV12 and ST36 is a valuable treatment option for FD.

In addition to similar therapeutic effects, significantly increased ALFF values in the left postcentral gyrus were found in all FD patients (combined group A and group B) after acupuncture treatment. The postcentral gyrus of the parietal lobe corresponds to the primary sensory cortex, receiving various sensations from the body (Yu et al., 2019; DiGuiseppi and Tadi, 2021). Multiple neuroimaging studies had previously confirmed the involvement of the postcentral gyrus in the central pathology of FD patients. For instance, a H_2_ (15) O-PET study found that FD patients demonstrated altered activity in the primary sensory cortex, including the postcentral gyrus, and that this altered activity corresponded to decreases in gastric distention (Van Oudenhove et al., 2010). Previous studies also found higher cerebral glucose metabolism in the postcentral gyrus in FD patients through 18 F-FDG PET-CT imaging (Zeng et al., 2011) and increased ALFF values in the postcentral gyrus through fMRI (Qi et al., 2020). These results indicated the role of the postcentral gyrus in the abnormal processing of gastrointestinal sensory signals. Furthermore, the participation of the postcentral gyrus in the integration of the regulatory effect of acupuncture on the gastrointestinal tract had been identified by several neuroimaging studies (Zeng et al., 2009; Zhou et al., 2013). Our previous studies indicated that acupuncture stimulation could decrease abnormally elevated glucose metabolism in the postcentral gyrus (Liu et al., 2012), and needling at ST36 could normalize the fMRI signals in the postcentral gyrus (Li et al., 2014). However, acupuncture does not always elicit brain responses in the postcentral gyrus, although brain responses have been observed in various acupuncture-related neuroimaging studies (Yan et al., 2005; Yang et al., 2014; Yeo et al., 2016; Zheng et al., 2018). This study indicated significant increases of ALFF values in the postcentral gyrus in all FD patients after acupuncture was performed, with increased ALFF values positively correlating with improvements in SID scores. The results indicated that the activity changes in the postcentral gyrus were related to acupuncture efficacy for FD.

### The Differences in Postcentral Gyrus Responses Caused by Needling at ST36 Versus CV12

This study aimed to investigate similar effects shared by different acupoints by differentiating response modes of two acupoints on the postcentral gyrus through rsFC analysis, as all patients exhibited changes in the functional activity of the postcentral gyrus after acupuncture treatment, with changes significantly positively correlating with the curative effect of acupuncture.

The results showed that needling at ST36 resulted in significantly increased rsFC between the left postcentral gyrus with precuneus, SFG, and MFG. It is well known that the precuneus is an important hub within the DMN (Brewer et al., 2011; Utevsky et al., 2014), which regulates the affective and sensory process together with the medial prefrontal cortex (mPFC) and amygdala (Vanner et al., 2016). The precuneus is involved in episodic memory retrieval (Cavanna and Trimble, 2006; Sajonz et al., 2010), appetite control (Scharmuller et al., 2012; Tuulari et al., 2015), appraisal of food (Winter et al., 2017), and reappraisal of the benefits of eating food (Yokum and Stice, 2013). A variety of studies have identified the structural and functional abnormalities of the precuneus in FD patients (Zeng et al., 2011; Liu et al., 2013). For example, Lee et al. (2018) observed that higher rsFC between the insula and precuneus was negatively correlated with FD symptoms, food craving, and depression while in a state of hunger. Our previous study also showed that DMN in FD patients may indeed undergo dysfunctional changes, with changes in DMN found to be related to FD symptom severity (Liu et al., 2013). More importantly, a number of studies have reported that acupuncture can reverse the disrupted DMN to achieve therapeutic effects in gastrointestinal disease (Bao et al., 2017; Sun et al., 2021a,b; Zhao et al., 2021). In these studies, needling at ST36 regulated the rsFC between the left postcentral gyrus with DMN. The results indicated that promoting self-regulation and adaptation *via* the DMN might be one of the functions achieved by needling ST36 for FD.

In contrast to ST36, CV12 elicited decreased rsFC values between the left postcentral gyrus and angular gyrus, caudate, MFG, and cerebellum in this study. The results indicated that needling at CV12 mainly regulated postcentral gyrus–RN circuitry in order to achieve treatment efficacy. The caudate nucleus and cerebellum, important components of the RN, play a crucial role in viscera activities (Allen et al., 1997; Ladabaum et al., 2001). Ladabaum et al. (2001) found significant activations of the bilateral caudate nucleus during proximal gastric dilation stimulation. Our previous results indicated increased gray matter volume in the right caudate (Liu et al., 2014), decreased gray matter density in the cerebellum (Zeng et al., 2013), and higher glycometabolism in the cerebellum (Zeng et al., 2011) in FD patients compared with healthy controls. In addition, higher glycometabolism in the cerebellum was decreased after acupuncture treatment (Zeng et al., 2015). These studies confirmed the structural and functional abnormalities in the caudate nucleus and cerebellum in FD patients, as well as the favorable regulatory effect of acupuncture on cerebellum activities. In fact, some researchers have found that the expectations of acupuncture efficacy may partly be based on the self-relevant phenomenon and self-referential introspection, which was often found to be related to activation of patient self-appraisal and RN (Pariente et al., 2005; Lundeberg et al., 2007).

In this study, performing acupuncture at ST36 and CV12 can affect the function of the postcentral gyrus, although the modes of influence are different. In addition, some studies focusing on peripheral nerve activity had found that the mechanisms of acupuncture at ST36 and CV12 to regulate gastrointestinal function were different. For example, one study had found that needling of the lower limb (ST36) caused gastrointestinal muscle contractions *via* the somatoparasympathetic pathway, while needling in the upper abdominal area (CV12) caused gastrointestinal muscle relaxation *via* the somatosympathetic pathway (Takahashi, 2006). Another study had indicated that electroacupuncture stimulation at the lower limb (ST36) but not in the abdomen (ST25) can promote the vagal–adrenal anti-inflammatory axis in mice (Liu et al., 2021). In summary, although acupoints in the different regions have similar therapeutic effects, the underlying mechanisms from central to peripheral were found to be relatively different.

## Limitations

There were some limitations in this study. Firstly, the sample size was relatively small. Secondly, we observed the changes in clinical symptoms and functional activities of FD patients at baseline and the end of 4 weeks of acupuncture treatment, but its long-term efficacy remains unclear due to a lack of follow-up studies. In the future, further studies are needed to assess the long-term efficacy and investigate the potential mechanism of “different acupoints exhibiting similar effects.”

## Conclusion

In conclusion, acupuncture at ST36 and CV12 had similar therapeutic efficacy in the treatment of FD, and the realization of therapeutic effect may be related to the modulation of the activity of the postcentral gyrus. Meanwhile, the modulatory pattern was relatively different. Namely, ST36 mainly affected the rsFC between the postcentral gyrus and DMN, while the CV12 mainly affected the rsFC between the postcentral gyrus and RN.

## Data Availability Statement

The raw data supporting the conclusions of this article will be made available by the authors, without undue reservation.

## Ethics Statement

The studies involving human participants were reviewed and approved by the Ethics Committee of the Affiliated Hospital of CDUTCM (approved number. 2014KL-028). The patients/participants provided their written informed consent to participate in this study.

## Author Contributions

FZ and QG: experimental design. ZH, PM, YQ, SYu, XL, TZ, LH, and JL: data collection. XD and TY: data analysis. XD: manuscript preparation. YC and SYi: manuscript revision. FZ: supervision. All authors contributed to the article and approved the submitted version.

## Conflict of Interest

The authors declare that the research was conducted in the absence of any commercial or financial relationships that could be construed as a potential conflict of interest.

## Publisher’s Note

All claims expressed in this article are solely those of the authors and do not necessarily represent those of their affiliated organizations, or those of the publisher, the editors and the reviewers. Any product that may be evaluated in this article, or claim that may be made by its manufacturer, is not guaranteed or endorsed by the publisher.

## Footnotes

1
http://rfmri.org/DPARSF
